# Antiproliferative Activity of Glycosaminoglycan-Like Polysaccharides Derived from Marine Molluscs

**DOI:** 10.3390/md16020063

**Published:** 2018-02-15

**Authors:** Abdullah Faisal Aldairi, Olanrewaju Dorcas Ogundipe, David Alexander Pye

**Affiliations:** School of Environment and Life Sciences, Cockcroft Building, University of Salford, Manchester M5 4WT, UK; A.Aldairi@edu.salford.ac.uk (A.F.A.); dorcasogundipe14@gmail.com (O.D.O.)

**Keywords:** marine mollusc, glycosaminoglycans, antiproliferative, anticancer, heparan sulphate

## Abstract

Despite the increasing availability of new classes of cancer treatment, such as immune- and targeted therapies, there remains a need for the development of new antiproliferative/cytotoxic drugs with improved pharmacological profiles that can also overcome drug resistant forms of cancer. In this study, we have identified, and characterised, a novel marine polysaccharide with the potential to be developed as an anticancer agent. Sulphated polysaccharides isolated from the common cockle (*Cerastoderma edule*) were shown to have antiproliferative activity on chronic myelogenous leukaemia and relapsed acute lymphoblastic leukaemia cell lines. Disaccharide and monosaccharide analysis of these marine polysaccharides confirmed the presence of glycosaminoglycan-like structures that were enriched in ion-exchange purified fractions containing antiproliferative activity. The antiproliferative activity of these glycosaminoglycan-like marine polysaccharides was shown to be susceptible to heparinase but not chondrotinase ABC digestion. This pattern of enzymatic and antiproliferative activity has not previously been seen, with either marine or mammalian glycosaminoglycans. As such, our findings suggest we have identified a new type of marine derived heparan sulphate/heparin-like polysaccharide with potent anticancer properties.

## 1. Introduction

Glycosaminoglycans (GAGs) are a complex family of polysaccharides found in both vertebrates and invertebrates. They bind to many proteins and mediate a diverse range of biological functions, including both cellular and physiological events [1]. These molecules are widely exploited as therapeutics, for example, hyaluronic acid (HA) and chondroitin sulphate (CS) are used to treat osteoarthritis, and of course heparin has been used as an anticoagulant and antithrombotic drug for more than 70 years [2]. However, no native mammalian GAGs have been shown to have direct cytotoxic or antiproliferative effects on cancer cells. There are five families of mammalian GAGs, each based on their repeating disaccharide units. These are heparan sulphate (HS) including the related molecule heparin, CS, dermatan sulphate (DS), keratan sulphate and the unsulphated HA. Heparan sulphate and CS/DS GAG chains are initially synthesised as an alternating backbone of *N*-acetylated amino sugar, either *N*-acetylglucosamine (GlcNAc) in HS or *N*-acetylgalactosamine (GalNAc) in CS/DS and glucuronic acid (GlcA), or its C5 epimer iduronic acid (IdoA). Epimerisation takes place to differing extents in HS and heparin and in the conversion of CS into DS. Further modifications occur by *N*-sulphation of the amino sugar (GlcNS) in HS/heparin and *O*-sulphation at various positions on the sugar units, commonly 2-*O*- and 6-*O*-positions [3]. The extent of both epimerisation and sulphation defines the interactions that take place between GAGs and their protein receptor partners [4]. Mammalian GAGs, in particular HS, are very heterogeneous in structure and are organised into subdomains that contain regions of high sulphation [5], which are important for interactions with proteins [6].

Marine invertebrates are a rich source of potential therapeutics and marine carbohydrates have been shown to have complex structures and biological activities [7]. Marine polysaccharides differ considerably from their mammalian counterparts, in terms of their structure and biological activities. As a result, they provide important new opportunities as carbohydrate based therapeutics [8]. The principle marine glycans are comprised of non-sulphated chitin, chitosan, sulphated GAGs, sulphated fucans and sulphated galactans. Marine polysaccharides from seaweed and algae have been shown to have anticancer activities but these are largely related to polysulphated fucans that are not directly members of the GAG family [8]. Sulphated fucans or fucoidans from marine sources are comprised of homogeneous unbranched polymers of (1–3) or (1–4) linked l-fucose units, which can be substituted with sulphate groups at positions C-2, C-4 and rarely at C-3. They may also be heterogeneous branched polymers with variable content of uronic acids and neutral sugars [9]. Single fucose units, or short fuco-oligosaccharide branches, can also be present on the polysaccharide backbone, usually at the C-4 position. Structural features within Fucoidans have been linked to many beneficial effects including anticancer [10,11,12], antiviral [13], anti-inflammatory [14] and antithrombotic activities [15]. GAG-like polysaccharides have been isolated from many marine species, including molluscs and in some instances, HS structures were found to be similar to heparin. Marine DS and CS have backbone structures comparable to mammalian GAGs but with differing sulphation patterns. Fucosylated CS and CS/DS hybrid chains have also been identified from marine sources [16,17]. Marine GAGs have not previously been linked with antiproliferative or cytotoxic effects on cancer cells.

In this study, we have shown that marine derived GAG-like polysaccharides from the common cockle (*Cerastoderma edule*) have antiproliferative activity on two cancer cell lines. Enzyme depolymerisation and structural analysis suggest that novel HS-like structures are responsible for the antiproliferative activity of the marine derived polysaccharides.

## 2. Results

### 2.1. Polysaccharide Isolation and Anticancer Activity

Numerous studies have portrayed the potential medicinal properties of marine polysaccharides, mainly from seaweed, and some have even demonstrated cytotoxic effects using both in vitro and in vivo models [18]. In this study, we show for the first time that marine polysaccharides isolated from molluscs, with structural similarities to mammalian GAGs, have in vitro antiproliferative effects on cancer cell lines.

**Figure 1:**
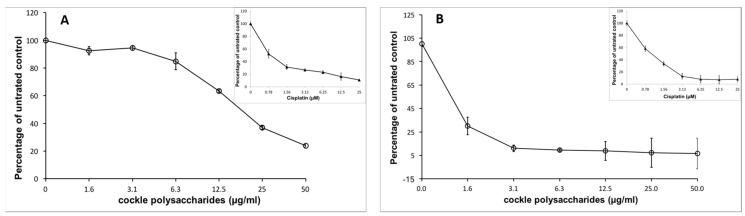
Antiproliferative activity of cockle polysaccharides on cancer cell lines. Two cancer cell lines K562 (**A**) and MOLT4 (**B**) were treated with increasing doses of cockle polysaccharides and cell viability was determined by MTT assay, as detailed in the “Materials and Methods” section. Inserts show effects of cisplatin treatment on viable cell number. Cells were cultured under standard conditions and maintained at 37 °C in a humidified 5% CO_2_ atmosphere. Cell viability is expressed as a percentage relative to untreated control cells. All experiments were conducted in triplicate and the IC_50_ values were calculated using non-linear regression analysis (GraphPad Prism 5.0).

Marine polysaccharides were isolated from common cockles, following a typical cetylpyridinium chloride extraction procedure [19]. The cockle polysaccharides were assessed against two leukaemia cell lines (K562 and MOLT4) for their ability to suppress cancer cell growth. The cockle polysaccharides showed considerable inhibition of cell proliferation with these cell lines, as determined by 3-(4,5-dimethylthiazole-2-yl)-2,5-diphenyltetrazolium bromide (MTT) assay (Figure 1).

Some batch-to-batch variability in IC_50_ values was seen with different isolations of cockle polysaccharides, presumably because of the complex heterogeneous mixture of polysaccharide chains present. Cisplatin was used in all assays, as a control for comparison of different preparations. Typical IC_50_ values were around 9 μg/mL and 1 μg/mL, respectively, for cell lines K562 and MOLT4.

### 2.2. Annexin V Apoptosis Detection and Cell Cycle Analysis

**Figure 2:**
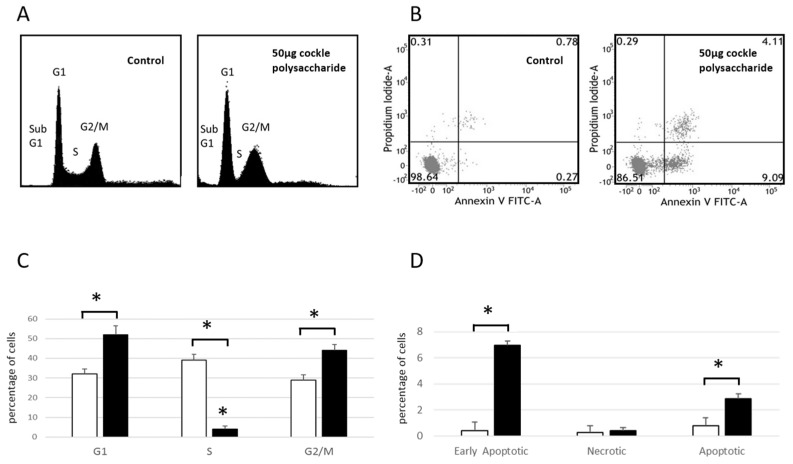
Cell cycle analysis and Annexin V apoptosis assay. MOLT4 cells were treated with 50 μg of cockle polysaccharides for 24 h then stained using Annexin V conjugated Fluorescein isothiocyanate (Annexin V-FITC) and/or PI. (**A**) Flow cytometry cell cycle analysis of PI stained cells with or without cockle polysaccharide treatment. A single representative experiment is shown. (**B**) Flow cytometry scatter plot of Annexin V-FITC/PI stained cells with or without cockle polysaccharide treatment. A single representative experiment is shown. (**C**) Quantitative cell cycle analysis as determined by PI staining and flow cytometry. The percentage of cells in each phase is shown for control (☐) and cockle polysaccharide treated (■) cells. Results are presented as the mean ± SD of three independent experiments. Statistical significance was determined using the two-tailed Student’s *t*-test. *p* < 0.05 was considered statistically significant (*). (**D**) Quantitative analysis of apoptosis as determined by Annexin V-FITC/PI staining and flow cytometry. The percentage of cells in each quadrant is shown for control (☐) and cockle polysaccharide treated (■) cells. Results are presented as the mean ± SD of three independent experiments. Statistical significance was determined using the two-tailed Student’s *t*-test. *p* < 0.05 was considered statistically significant (*).

Cell Cycle analysis of MOLT-4 cells (Figure 2A,C) showed an increase in cell numbers in both G1 and G2/M phases after 24 h exposure to cockle polysaccharides, accompanied by a lowering of cells in S-phase. These data, and the presence of cells in sub G1, indicate a complex mechanism of cell death, following treatment of MOLT-4 cells with cockle polysaccharides, potentially mediated via apoptosis. Flow cytometry and Annexin V/propidium iodide (PI) staining, was used to further investigate the mechanism of cell death following cockle polysaccharide treatment (Figure 2B,D). Leukaemia cell line MOLT-4 showed small but significant increases in late apoptotic (Annexin V^+^/PI^+^) cells when treated with cockle polysaccharide, However, early apoptotic (Annexin V^+^/PI^−^) showed the biggest change in cell populations after a 24 h incubation. Necrotic (Annexin V^−^/PI^+^) cells showed little change over untreated controls. The results shown in Figure 2 clearly implicate apoptosis as the most likely cause of the cytotoxicity seen following treatment by cockle polysaccharides. However, primary necrosis cannot be ruled out. Data from K562 cells were not obtained, as their inherent tendency to aggregate proved problematic in the flow cytometry studies.

### 2.3. Effect of Enzymatic Degradation on Cockle Polysaccharide Anticancer Activities

**Figure 3:**
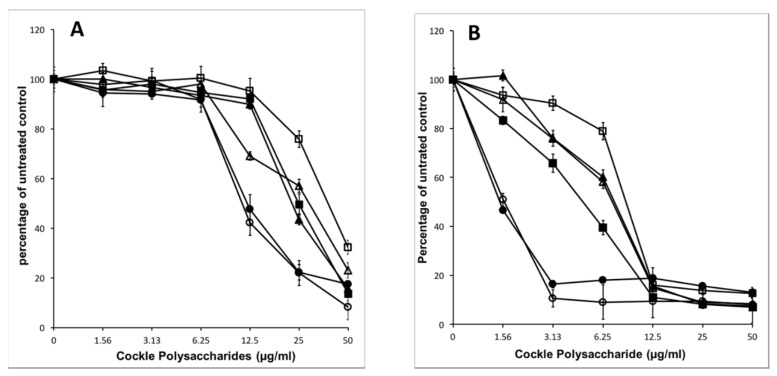
Effect of heparinase (I, II, and III) and chondroitinase ABC enzymatic degradation on cockle polysaccharide antiproliferative activity. Sensitivity of the cockle polysaccharide antiproliferative activity to enzymatic degradation was determined by MTT assay. Antiproliferative activity of cockle polysaccharides on K562 cells (**A**) and MOLT4 cells (**B**), with and without heparinase or chondrotinase ABC digestion. Intact cockle polysaccharides (◯), heparinase I treated (∆), heparinase II treated (▲), heparinase III treated (■), heparinase I, II, and III treated (□) and chondroitinase ABC (●). The data are presented as the percentage of viable cells following treatment with cockle polysaccharides, relative to untreated control. All experiments were conducted in triplicate and the results are shown as the mean ± the SD. Cells were cultured in suspension and maintained at 37 °C in humidified 5% CO_2_ atmosphere.

The extraction procedure used, whilst designed to enrich the content of sulphated GAG or GAG-like structures, will also bring contamination from other marine polysaccharides. These non-GAG polysaccharides may contribute solely, or in part, to the observed antiproliferative activity of the cockle polysaccharides. Enzymatic treatment of the cockle polysaccharides by heparinases I, II, III and chondroitinase ABC was used to investigate any link between typical GAG-like structures and the observed antiproliferative activity. Figure 3 demonstrates that there is no appreciable change in antiproliferative activity when cockle polysaccharides are incubated with chondroitinase ABC. Heparinase treatment (individually and in combination) however, did lead to a significant loss of biological activity, with an approximate increase in IC_50_ values for combined heparinases I, II, III of around 4- and 8-fold for K562 and MOLT4 cell lines respectively. There was little difference between the individual heparinases enzymes, however in replicates heparinase II, was consistently more effective at reducing the antiproliferative effects of the cockle polysaccharides. Despite the significant loss in activity, inhibition was not entirely destroyed by heparinase treatment, even when used in combination. This suggest that a proportion of resistant disaccharide linkages may exist and that resistant fragments are of sufficient size to still function as an antiproliferative. Taken together, the data suggest that HS/heparin like GAG structures are key contributors to the antiproliferative activity of the cockle polysaccharides, with CS-like GAGs contributing little to the anticancer affect.

### 2.4. Comparisons of Marine GAG Antiproliferative Activity with Mammalian GAGs

**Figure 4:**
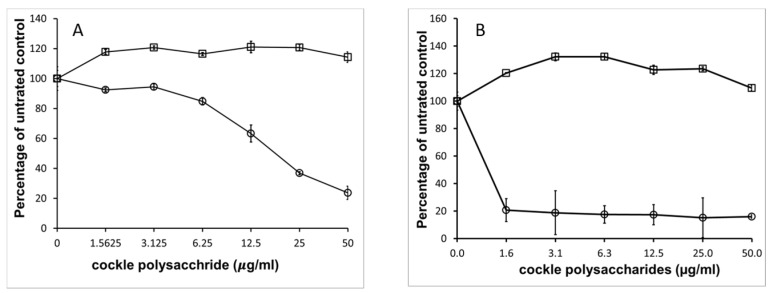
Comparison of mammalian GAGs and cockle polysaccharides antiproliferative activity. Differences in biological activities of mammalian GAGs and cockle polysaccharides on K562 (**A**) and MOLT4 (**B**) cell lines was assessed by MTT assay, cockle polysaccharides (◯), mammalian HS (☐). Data are presented as the percentage of viable cells following treatment with cockle polysaccharides, relative to untreated control. All experiments were conducted in triplicate and the results are shown as the mean ± the SD. Cells were cultured in suspension and maintained at 37 °C in humidified 5% CO_2_ atmosphere.

The potent antiproliferative effect of cockle polysaccharides was surprising, as the anticancer activity appeared to be linked to the presence of mammalian HS/heparin-like structures, which to our knowledge have never shown direct antiproliferative effects on cancer cell lines in vitro. Further investigation confirmed that mammalian derived GAGs (porcine mucosal) had no antiproliferative activity in our MTT assay system. Figure 4 shows that no measurable IC_50_ could be determined for mammalian HS, with either cancer cell line, yet the cockle polysaccharides continued to show significant antiproliferative activity. In fact, at low concentrations mammalian HS showed slight stimulatory activity on cell growth. The results suggest that, if HS-like-GAG chains are involved in the antiproliferative activity of cockle polysaccharides, then they must contain unique structural features not found in typical mammalian GAGs.

### 2.5. Disaccharide Analysis of GAGs

Disaccharide analysis was used solely to confirm the presence of typical unsaturated HS and CS/DS disaccharides, following treatment of the cockle polysaccharides with heparinase I, II and III or chondroitinase ABC and was not part of any detailed structure/activity study.

Initial analysis of the HS/heparin disaccharide compositions, produced by combined heparinase I, II, III digestion, showed that susceptible chains within the cockle polysaccharide preparations did contain the major disaccharide species found in mammalian HS and heparin. Care must be taken in attributing total HS disaccharide compositions to these chains, as activity data (Figure 3) suggest that the combined heparinase I, II, and III digests are possibly incomplete. The major differences between disaccharides derived from heparinase digestion of mammalian HS and the cockle polysaccharide (Table 1) were the low levels of unsulphated disaccharide ∆HexA-GlcNAc and the high levels of the disulphated disaccharide ∆HexA-GlcNS(6S) released from the cockle polysaccharides. Other disaccharides were obtained at similar levels to those seen with many typical mammalian HS types. The heparinases failed to generate high quantities of the trisulphated disaccharide ∆HexA(2S)-GlcNS(6S) from the crude cockle polysaccharides, indicating that the HS-like cockle polysaccharides chains are more akin to mammalian HS than heparin. CS/DS disaccharide analysis was carried out, despite the fact that chondrotinase ABC treatment of cockle polysaccharides failed to show any significant loss of antiproliferative activity. The principle disaccharide components produced by chondroitinase ABC digestion were ∆HexA-GalNAc(4S), ∆HexA-GalNAc(6S) and ∆HexA-GalNAc(4S)(6S). The results clearly show that CS/DS-like chains are also present in the cockle polysaccharide preparations.

Disaccharide analysis data support the initial suggestion that the reduction in antiproliferative activity, following treatment of cockle polysaccharides with heparinases, was due to their breakdown. Resulting in the release of unsaturated disaccharides commonly found in mammalian HS/heparin. The lack of activity seen with the mammalian GAGs in the MTT assay, and the presence of heparinase resistant fragments within the cockle polysaccharides, suggests that the active HS-like-chains differ considerably from their mammalian counterparts.

### 2.6. Ion-Exchange Chromatography

**Figure 5:**
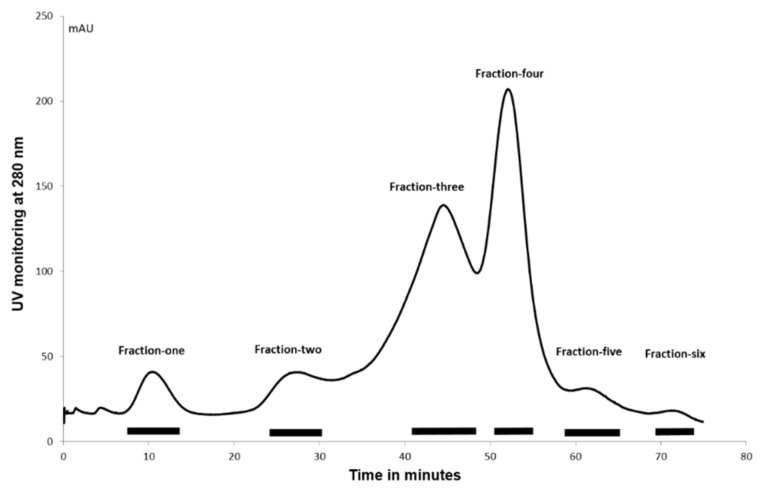
Ion-exchange chromatography of cockle polysaccharides. Cockle polysaccharides were applied to a DEAE-Sepharose column and eluted using a 0–1.5 M NaCl gradient over 70 min. Peaks were pooled as indicated by the bars shown, lyophilised and Fractions 1–6 stored at −20 °C for further analysis.

The complexity of the cockle polysaccharide mixtures limited our ability to identity particular structural features responsible for their antiproliferative effects. Clearly, the cockle polysaccharides contain both HS and CS/DS-like chains and probably other non-GAG chains. Consequently, ion-exchange chromatography was performed to evaluate further the structure/activity features of the components contained within the unfractionated cockle polysaccharides preparations. The elutant was monitored at 280 nM to detect the peptide fragments still attached to the polysaccharide chains following the initial protease digestion. A typical elution profile is shown in Figure 5, with six peaks generally occurring. The absorbance measured under each peak was broadly comparable to the dry weights of polysaccharides obtained after desalting and lyophilisation.

### 2.7. MTT Assay of Ion-Exchange Purified Cockle Polysaccharide Fractions

**Figure 6:**
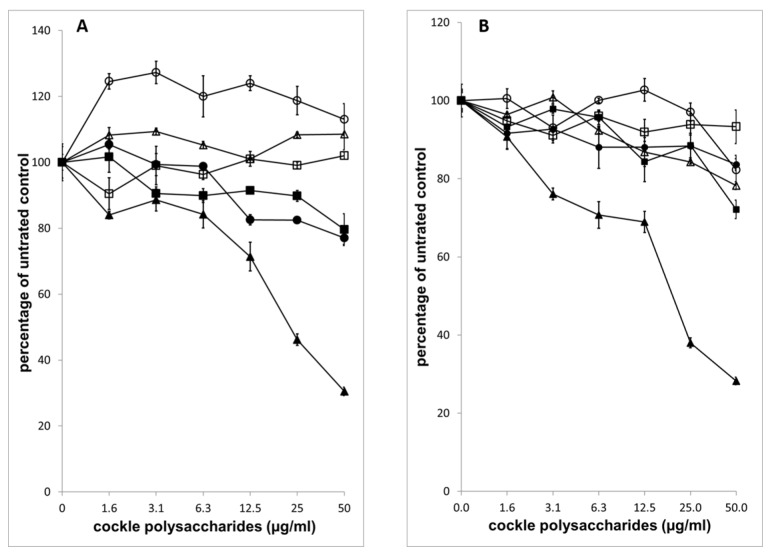
Antiproliferative properties of the ion-exchange purified cockle polysaccharide fractions. Measurement of antiproliferative activity of ion-exchanged purified fractions on K562 (**A**) and MOLT4 (**B**) cells was achieved by MTT assay. Fraction 1 (◯), Fraction 2 (☐), Fraction 3 (∆), Fraction 4, (■), Fraction 5 (▲) and Fraction 6 (●). The data are presented as the percentage of viable cells following treatment with cockle polysaccharides, relative to untreated control. All experiments were conducted in triplicate and the results are shown as the mean ± the SD. Cells were cultured in suspension and maintained at 37 °C in humidified 5% CO_2_ atmosphere.

Antiproliferative activity of ion-exchange purified cockle polysaccharides against K562 and MOLT4 cells (Figure 6) showed that most of the antiproliferative activity was seen with Fraction 5, with negligable antiproliferative activity observed with Fractions 1–3. The elution profile from the ion-exchange column suggests that the low levels of activity observed with Fractions 4 and 6 are probably due to cross contamination from Fraction 5. The results indicate that the antiproliferative cockle glycans are a relatively minor component of the unfractionated mixtures (approximately 1–2% of the total dry mass applied to the column).

### 2.8. Disaccharide Analysis of Ion-Exchange Purified Polysaccharides

Disaccharide analysis of the ion-exchange purified fractions was used to identify further the nature of the different glycan chain types within the cockle polysaccharide preparations and to confirm the role of HS-like-GAG structures in the antiproliferative activity. Table 2 shows the relative distribution of unsaturated disaccharides and the amounts produced when each fraction was treated exhaustively with a combination of heparinases I, II and III. The total weight of disaccharides produced from each microgram of sample digested showed that only Fraction 5 produced a significant amount of unsaturated disaccharides. Fractions 2, 3, 4 and 6 produced approximately 30-, 150-, 30-, 6- and 5-fold less disaccharide than Fraction 5, respectively, suggesting that the early eluting fraction contain mainly non HS-like chains. The composition of Fractions 5 and 6 showed the presence of all the major disaccharides found in mammalian HS/heparin. The enrichment of the trisulphated disaccharide ∆HexA(2S)-GlcNS(6S) and the disulphated disaccharides ∆HexA(2S)-GlcNS with an approximate four-fold and three-fold increase, respectively, in comparison to the unfractionated cockle polysaccharides (Table 1), likely contributes significantly to the later elution of these chains from the ion-exchange column. Fraction 1 appears to generate a higher proportion of sulphated disaccharides than any other fraction; however, the quantity, in μg, of disaccharides produced is very small in comparison to the total material digested. In fact, there is little correlation between the increase in the number of sulphates per disaccharide produced from heparinase digestion and the elution position of Fractions 1–4. This is likely linked to the limited digestion of the glycan chains contained within them. 

Disaccharide analysis of each fraction, following chondroitinase ABC digestion (Table 3), also showed early eluting fractions lacked correlation between the sulphate content of the resultant disaccharides and the fractions elution position. Again, this is likely linked to the incomplete nature of the digests, as confirmed by the quantity, in μg, of disaccharides released by digestion. The later eluting fractions produced disaccharides compositions close to those seen for the unfractionated cockle polysaccharides (Table 1). The later eluting Fractions 5 and 6 contain CS-like-GAG chains, however Figure 3 suggests that these chains do not contribute to the antiproliferative activity of Fraction 5.

Taken together, the data imply that the majority of heparinases and chondrotinase ABC susceptible linkages, and therefore GAG-like structures, are present in the later eluting fractions from the ion-exchange separation. The production of a significant quantity of HS disaccharides, from the highly active Fraction 5, confirms the link between HS-GAG-like structures and the antiproliferative activity of the cockle polysaccharides. However, the residual activity of the heparinase resistant chains must also be taken into account when judging any structure/activity relationships.

### 2.9. Monosaccharide Analysis of Ion-Exchange Purified Polysaccharides

The lack of information regarding the identity of the uronic acid moieties, using the method of disaccharide analysis employed in this study, means that we cannot identity potentially important structural features within the GAG-like chains. We are also missing structural detail on the content of non-GAG-like components of the cockle polysaccharide preparations. These issues can be clarified by total acid hydrolysis and monosaccharide analysis of both the unfractionated cockle polysaccharides and the ion-exchanged purified fractions. Analysis of the unfractionated cockle polysaccharide preparations (Table 4) showed the presence of significant quantities of the monosaccharides, GalNH_2_, GlcNH_2_, and GlcA, which are typically found following acid hydrolysis of mammalian and marine GAG chains. Interestingly, there was no detectable IdoA component. Fucose made up about 10% of the monosaccharides produced and could be derived from fucoidan-like or fucosylated CS glycans. The neutral sugars glucose (Glc) and galactose (Gal) were by far the most predominant monosaccharaides present in the unfractionated cockle polysaccharide preparations and represent components typically found in marine *N*- and *O*-linked glycans. Mannose represented less than 4% of the monosaccharides released by hydrolysis, and was predominantly found in Fractions 2 and 6. The data support the presence of GAG-like chains, as was indicated by both the susceptibility of cockle polysaccharides to heparinase/chondroitinase ABC digestion, and the production of a range of unsaturated disaccharides, typically found in mammalian GAG chains. Analysis of ion-exchange purified fractions showed enrichment of the GAG related monosaccharides GalNH_2_, GlcNH_2_, and GlcA in the later eluting fractions (Fractions 3–6) and critically the antiproliferative Fraction 5, which also contained the highest amount of GlcA.

Overall, the data indicate the presence of a diverse range of glycan chains, including GAG-like structures, within the unfractionated cockle polysaccharide preparations. The components found in the active Fraction 5 are clearly GAG-like in nature. The lack of IdoA is an interesting observation, yet this does not, on its own, explain the unique activity of the cockle derived HS-like GAG chains. The complexity of the cockle polysaccharide preparations, even within the ion-exchange purified fractions, and the limitations of the compositional analysis tools used in this study make it difficult to determine the exact structural differences between the antiproliferative cockle HS-like GAG chains and their inactive mammalian counterparts. We also need to consider the nature of the antiproliferative activity that remains following extensive heparinase digestion.

## 3. Discussion

We have shown for the first time that marine derived polysaccharides, with proven anticancer activity, are susceptible to the effects of heparinase digestion. The results, in conjunction with the lack of antiproliferative activity of mammalian GAGs, suggest that atypical GAG structures are present in marine polysaccharides from mollusc sources. These unique structures can be directly linked, at least in part, to the antiproliferative activity seen on human cancer cell lines treated with cockle polysaccharides.

Anti-metastatic and antiproliferative activity of marine derived polysaccharides has previously been observed from a variety of sources, such as algae, ascidians, euechinoidea, molluscs and bacteria [20]. The active molecules identified to date are classes of sulphated polysaccharides, such as sulphated fucans and sulphated galactans, which are defined as non-GAG glycans. They differ from typical mammalian GAG family members in several ways, including their relative homogeneity, in terms of their monosaccharide compositions and distribution. Their most dramatic structural differences lie in the type of monosaccharide building blocks that make up these chains. Sulphated fucans are polymers of fucopyranso units, which may contain variable amounts of neutral monosaccharides and uronic acids and are predominantly classed as fucoidans [9,21], whereas sulphated galactans are almost exclusively composed of Gal units [22]. None of the glycosidic linkages within these marine polysaccharides has any reported susceptibility to the enzymes used in this study. Hence, the reduction in antiproliferative activity we observed following heparinase digestion of the cockle polysaccharides suggests that the anticancer activity of the intact chains is linked to the presence of archetypal mammalian GAG disaccharide structures. Clearly, this is divergent with the literature that to date has exclusively linked anticancer activity of marine polysaccharides to non-GAG glycans. Some heterogeneity has been reported for some of the sulphated fucans [23], and galactans [24]. However, none of the variations was connected to the presence of mammalian like GAG disaccharides.

We have not yet considered previously discovered marine polysaccharides that are closely related to traditional mammalian GAG structures, such as fucosylated CS, dermatan sulphate-like polysaccharides, GlcA containing HS and highly sulphated hybrid heparin/HS chains. Despite their widespread presence, no antiproliferative activity, on cancer cell lines, has previously been attributed to these polysaccharides classes. Fucosylated CS from *holothuroidea* has been reported to be insensitive to chondroitinase ABC digestion, without prior removal of fucose residues [25] and highly sulphated dermatan-like polysaccharides from tunicates have been shown to be sensitive to chondroitinase ABC digestion [26,27]. In our study, unsaturated disaccharides were produced by chondroitinase ABC digestion of cockle polysaccharides, although this had no effect on the antiproliferative activity. We have not confirmed the presence of fucosylated CS or dermatan like polysaccharides in the active cockle polysaccharide fractions. As such, we cannot rule out a contribution by these types of structure to the observed antiproliferative effects. However, the absence of IdoA in the monosaccharide analysis suggest that dermatan-like structures are unlikely to be present in the cockle polysaccharide preparations.

The heparin/HS type of polysaccharide chains identified from marine sources include a report of a unique hybrid heparin/HS polysaccharide found in the head of shrimp. These marine heparin/HS hybrid GAGs were extensively degraded by heparinase enzymes [28]. The GlcA containing HS-like GAGs derived from the mollusc *Nodipecten nodosus* represents another heparinase sensitive structure that may be linked to the antiproliferative activity of the cockle polysaccharides [29]. Although no antiproliferative activity has been reported, to date, with either of these types of glycan chains, the cockle polysaccharides did loose significant antiproliferative activity, following heparinase degradation (Figure 3). This observation might be linked to the presence of similar hybrid heparin/HS or GlcA-rich HS structures in the cockle polysaccharide preparations. However, appreciable antiproliferative activity did remain, even after extensive heparinase treatment of the unfractionated cockle polysaccharides. Suggesting that other atypical GAG structures are potentially involved in the antiproliferative activity. A reductionist assessment of the structural features within these heparinase resistant sequences represents an attractive approach that may well lead to a more thorough understanding of precise structural features required to facilitate that anticancer activity seen in this study.

Disaccharide analysis was employed to identify compositional differences between the building blocks of known marine/mammalian GAGs and the antiproliferative chains within cockle-derived polysaccharides. Our data showed that heparinase I, II, III treatment of cockle polysaccharides liberated all of the most abundant unsaturated disaccharide types typically observed in mammalian GAGs. The rare 3-*O*-sulphated disaccharides were not detected in any of the cockle polysaccharide samples, using the methods described here. The most significant feature of the analysis appeared to be related to the overall levels of *N*-sulphated disaccharides produced by heparinase digestion, which accounted for approximately two thirds of the disaccharide units obtained from the unfractionated cockle polysaccharide preparations. The antiproliferative ion-exchange purified, Fraction 5, yielded in access of 80% *N*-sulphated disaccharides, as opposed to the much lower levels, typically around 50%, produced by combined heparinase digestion of mammalian HS. For example, porcine mucosal HS contains approximately 40% *N*-sulphated disaccharides [30]. Mammalian heparins typically have higher *N*-sulphate levels, averaging around 85% of the disaccharide units [31]. The other notable differences are in the levels of the disaccharides ∆HexA(2S)-GlcNS and ∆HexA-GlcNS(6S) generated by combined heparinase digestion of some of the ion-exchange purified fractions, making up 24.4% and 19.4% of the disaccharides liberated from the active Fraction 5, respectively, as opposed to the percentage of these disaccharide in porcine mucosal heparin (0.9% and 14.6%, respectively), porcine mucosal HS (10.4% and 3.9%, respectively), bovine kidney (2.9% and 5.1%, respectively) and rat liver HS (14.5% and 6.2%, respectively) [30,31]. Despite Fraction 5 showing significant antiproliferative activity in comparison to the inactive porcine mucosal HS, it seems unlikely that the increased presence of these disaccharides is solely responsible for the differences in antiproliferative activity observed in this study. In fact, the analysis of the disaccharides liberated from the unfractionated cockle polysaccharides, and the active Fraction 5, by heparinase gave no definitive clues to the differences in biological activity seen between the marine sourced cockle polysaccharides and mammalian GAGs. Overall, the lack of antiproliferative activity of mammalian GAGs suggest that a radically different type of HS-like structure may be present in the cockle polysaccharides.

Analysis of the monosaccharide composition of the cockle polysaccharides, again failed to give a conclusive insight into the specific glycan structures that might be facilitating the antiproliferative activity of these glycan chains. The monosaccharide analysis did confirm that GAG-like structures where enriched in the unfractionated cockle polysaccharides and the active Fraction 5. However, this compositional detail and the complete absence of IdoA in the cockle polysaccharides does not yet represent sufficient evidence to conclude the exact nature of the antiproliferative glycan structures.

It might be suggested that the antiproliferative effects observed in this study, simply represent a non-specific structure/activity relationship linked to the very high sulphate content of the cockle polysaccharides and the active Fraction 5. To the authors knowledge no studies have demonstrated any direct antiproliferative effects of mammalian heparins on cancer cells, using in vitro MTT assays, despite their high sulphation levels. A report has been published, in which a clinical preparation of low molecular weight heparin showed slight inhibition of a human lung adenocarcinoma A549 cell line by MTT assay. However, 50% inhibition was never achieved at the concentrations used [32]. In addition, the low molecular weight heparins used failed to induce apoptosis, unlike the cockle polysaccharides (Figure 2). Intact high molecular weight porcine mucosal heparins, used in our study, failed to show any inhibition of cancer cell growth (results not shown). In addition, the activity profile for the ion-exchange purified fractions showed that the more heavily sulphated Fraction 6 had significantly less antiproliferative activity than the earlier eluting Fraction 5. However, both fractions produced HS/heparin like disaccharides following heparinase treatment and Fraction 6 had a higher overall sulphate content (Table 2). Taken together, these results suggest that the high levels of sulphation found in these cockle polysaccharides are not the principle reason for their antiproliferative activity. Hence, it is likely that specific sequences within the polysaccharides are mediating their biological activities in a distinct manner.

Marine polysaccharides continue to attract great interest as molecules with the potential for therapeutic development. In this study, we have identified antiproliferative and apoptotic activity in sulphated polysaccharides isolated from a marine mollusc. These novel GAG-like chains differ significantly from their mammalian counterparts, in terms of structure and anticancer activity, and have the potential to be developed into new a new class of cancer therapeutics.

## 4. Materials and Methods

### 4.1. Extraction of Sulphated Polysaccharides

Polysaccharides were extracted from common cockle (*Cerastoderma edule*), obtained from the Irish Sea, British Isles, using a standard protocol [19]. Shells were removed and the entire soft body tissue was defatted by incubation with acetone for 72 h. Defatted tissue was left to dry for 24–48 h then ground to a fine powder. The powder (4 g) was suspended in 40 mL of 0.05 M of sodium carbonate pH 9.2, and 2 mL of Alcalase enzyme (Merck, Millipore, Watford, UK) added. Samples were then incubated at 60 °C for 48 h with constant agitation at 200 rpm. The mixture was then cooled at 4 °C and trichloroacetic acid (Sigma-Aldrich, Gillingham, UK) added to a concentration of 5% (*w*/*v*) and left for 10 min. Precipitated peptides were removed by centrifugation (5000× *g* for 10 min). The supernatant was retained and three volumes of 5% (*w*/*v*) potassium acetate in ethanol was added to one volume of supernatant and the mixed solution left overnight at 4 °C. Precipitated cockle polysaccharide chains were recovered by centrifugation (5000× *g* for 30 min) and the pellet washed with absolute alcohol. The recovered precipitate (1 g) was then dissolved in 40 mL of 0.2 M NaCl solution and centrifuged (5000× *g* for 30 min) to remove any insoluble material. Cetylpyridinium chloride (Sigma-Aldrich) (0.5 mL of a 5% (*w*/*v*) solution) was added to the supernatant and the precipitate formed, recovered by centrifugation (8000× *g* for 30 min). The precipitate was subsequently dissolved in 10 mL of 2.5 M NaCl solution, followed by the addition of 5 volumes of ethanol. The precipitated cockle polysaccharide chains were recovered by centrifugation (8000× *g* for 30 min) before being dialysed against water for 72 h. The dialysate was lyophilized to obtain a white powder containing approximately 2.0 mg of cockle polysaccharides.

### 4.2. Maintenance of Cell Lines

Human cancer cell lines K562 (Chronic myelogenous leukaemia) and MOLT-4 (acute T lymphoblastic leukaemia) were grown in RPMI-1640 medium containing 1 g/L glucose (Lonza Group Ltd., Basel, Switzerland), supplemented with 10% (*v*/*v*) inactivated FBS (Labtech International Ltd., Heathfield, UK), 2 mM l-glutamine (Labtech International Ltd.), 100 Units/mL penicillin and 100 ug/mL streptomycin (Labtech International Ltd.). All cell lines were maintained in 25 cm^2^ flasks under a humidified atmosphere of 95% air and 5% CO_2_ at 37 °C.

### 4.3. Cell Viability Assay 

Cell viability was assessed using an MTT (3-(4,5 dimethylthiazol2-yl)-2,5 diphenyltetrazolium bromide) (Sigma-Aldrich) assay. Cells were seeded at a density of 5 × 10^4^ cells/well and cultured overnight in 96-well plates containing 100 µL of culture medium prior to treatment with polysaccharide samples. Treatment was conducted for 96 h following addition of various concentrations of cockle polysaccharides and mammalian GAGs (Celsus, Cincinnati, OH, USA) (0–50 µg/mL), with cisplatin (0–25 mM) as a positive control. At the end of the incubation period, 50 µL MTT solution (5 mg/mL in PBS) was added to each well and incubated for 3 h at 37 °C. Next, 200 μL of DMSO was added to each well and the plates agitated to dissolve the formamazan crystal product. The amount of MTT converted to formazan is indicative of the number of viable cells. The plates were gently agitated until the colour reaction was uniform and the absorbance was measured at 570 nm using a multi-well plate reader. The cell viability effects from exposure of cells to each concentration of crude cockle polysaccharides, commercial mammalian GAGs and cisplatinum (Sigma-Aldrich) were analysed as percentages of the untreated control cell absorbance. The average cell survival obtained from triplicate determinations, at each concentration, was plotted as a dose–response curve. The IC_50_ values were calculated using nonlinear regression analysis (GraphPad Prism 5.0, La Jolla, CA, USA)

### 4.4. Cell Cycle and Annexin V Apoptosis Assay

Cells were seeded into sterile 6-well plates at 5 × 10^5^/mL, and incubated with or without cockle polysaccharides at 37 °C. Cells were harvested at designated time intervals, centrifuged at 2000× *g* for 5 min, washed twice with cold PBS and resuspended in 1 × binding buffer (0.1 M HEPES (Sigma-Aldrich), 1.4 M NaCl and 25 mM CaCl_2_) at a concentration of 1 × 10^6^ cells/mL. Cells (100 µL) were then transferred into 5 mL culture tube and stained with Annexin V-FITC (5 µL) and propidium iodide (PI) (Sigma-Aldrich) (10 µL). The stained cells were gently vortex for few seconds and incubated in the dark for 15 min at room temperature. Binding buffer (400 µL) was added to each tube prior analysis by flow cytometer (BD FACSVerse, Franklin Lakes, NJ, USA). Prior to flow cytometry cell cycle analysis, cells (100 µL) were centrifuged, washed in PBS three times and incubated at room temperature with 50 µL of Ribonuclease A (RNase) (50 mg/mL) before staining with a solution containing 50 µg/mL propidium iodide (PI) for 30 min in the dark.

### 4.5. Enzymatic Digestion 

Polysaccharide samples were digested by heparinase or chondroitinase ABC prior to MTT assay, as follows. Enzyme buffer comprised of 10 mM calcium acetate and 50 mM sodium acetate pH 7.0 containing 30 mIU of heparinase I, II, III (Grampian Enzymes, Aberdeen, UK) separately or in combination was added to solutions of 100 µg of cockle polysaccharides in enzyme buffer. Chondroitinase ABC (Grampian Enzymes, Aberdeen, UK) (30 mIU) in enzyme buffer was added to 100 µg cockle polysaccharides and incubated for 24 h at 37 °C. The enzyme digests were monitored spectrophotometrically at 232 nM and the reactions terminated by heating at 100 °C for 5 min.

### 4.6. Ion-Exchange Chromatography

Crude cockle polysaccharide preparations were fractionated by anion exchange chromatography, using an FPLC system (Pharmacia, Stockholm, Sweden). Samples were applied to an ion-exchange column (16 × 200 mm), packed with 10 mL of DEAE-Sepharose (GE Healthcare, Little shallot, UK). Cockle polysaccharides were eluted using a linear 0–1.5 M NaCl gradient in 50 mM sodium phosphate buffer pH 7.0 over 70 min at a flow rate of 1 mL/min. Absorbance was monitored at 280 nm, 1 mL fractions were collected. and pooled as indicated. 

Pooled fractions, corresponding to the peaks in the elution profile were dialysed extensively against water using 14 kDa molecular weight cut-off tubing (Scientific Laboratory Supplies, Nottingham, UK). After dialysis, peaks were lyophilised, and stored at −20 °C.

### 4.7. HPAEC-PAD Monosaccharide Analysis 

HPAEC-PAD was preformed to determine monosaccharide composition, using a Dionex ICS-3000 system (Sunnyvale, CA, USA) [33]. Crude cockle polysaccharides (50 µg) or pooled peaks from the ion-exchange chromatography separation, were dissolved in 200 μL of MilliQ water. The mixture was then hydrolysed using 1 mL of trifluoroacetic acid at 100 °C for 6 h to ensure complete hydrolysis of polysaccharides to monosaccharides. Monosaccharide samples were next centrifuged at 2000 rpm for 2 min. Acid was removed using a dry nitrogen flush after addition of 50 μL of 50% (*v*/*v*) aqueous isopropyl alcohol. Monosaccharide samples (20 µg) were applied to a Dionex CarboPac PA1 column, 4 mm × 250 mm, using a 4 µm and 4 mm × 50 guard column at a flow rate 1 mL/min. Monosaccharides were eluted using a gradient formed from three solvents: water as solvent A, 100 mM NaOH with 5 mM NaOAc as solvent B, and 100 mM NaOH with 250 mM NaOAc as solvent C. Peaks were detected using pulsed amperometric detector with standard quad waveform. 

### 4.8. Disaccharide Analysis

The cockle polysaccharide samples were fractionated by ion-exchange chromatography prior to disaccharide analysis as described previously.

Crude cockle polysaccharide or ion-exchange fractionated polysaccharides were incubated with a mixture of 10 mIU each of heparinases I, II, and III in lyase buffer (100 mM sodium acetate pH 7.0, containing 0.1 mM calcium acetate). Samples were incubated overnight at 37 °C and the reaction terminated by heating to 100 °C for 5 min. The samples were passed through a 10 K MWCO spin filter then dried before further analysis. GC-MS and isotopic aniline tagging (GRIL-Glycan Reductive Isotope Labelling) was used for composition analysis and mass detection of disaccharide yield from heparinase treated cockle polysaccharides [34]. 15 μL of ^12^C_6_ labelled Aniline and 15 μL of 1 M sodium cyanoborohydride, freshly prepared in DMSO:acetic acid (7:3, *v*/*v*), was added to 8 ρmol of dried heparinase derived disaccharides. Reactions were carried out at 37 °C for 16 h, and then dried in a centrifugal evaporator. The dried samples were prepared for LCQ-MS analysis by resuspending in running buffer (8 mM acetic acid, 5 mM dibutylamine (DBA)) followed by centrifugation at 14,000× *g* for 7 min. The supernatant (5 μL) was spiked with an 8 ρmol solution of unsaturated disaccharide standards tagged with^13^C_6_ aniline (2 μL) and the sample made up to 10 μL with running buffer. Aniline isotopic and non-isotopic disaccharides were separated on a C_18_ reversed-phase column (0.46 cm × 25 cm, Vydac). The solvent system used to elute the samples was 100% buffer A (8 mM acetic acid, 5 mM DBA) for 10 min, 17% buffer B (70%, methanol 8 mM acetic acid 5 mM DBA) for 15 min, 32% buffer B for 15 min, 40% buffer B for 15 min, 60% buffer B for 15 min, 100% buffer B for 10 min and 100% buffer A. Ions of interest were detected in negative ion mode and the capillary temperature and spray voltage were kept at 140 °C and 4.75 kV, UV detection was at 232 nm.

## Figures and Tables

**Table 1:** Disaccharide analysis of crude unfractionated cockle polysaccharides. 1: Data are presented as a percentage of the moles of CS/DS and HS unsaturated disaccharides produced by chondroitinase ABC and heparinase I, II, and III digestion.

**HS Disaccharides**	**Disaccharides Produced (%) ^1^**
∆HexA-GlcNAc	26.6
∆HexA(2S)-GlcNAc	0.0
∆HexA(2S)-GlcNH_2_	0.0
∆HexA-GlcNAc(6S)	4.1
∆HexA(2S)-GlcNAc(6S)	4.0
∆HexA-GlcNS	25.5
∆HexA(2S)-GlcNS	9.5
∆HexA-GlcNS(6S)	24.7
∆HexA-GlcNH_2_(6S)	0.0
∆HexA(2S)-GlcNS(6S)	5.6
∆HexA(2S)-GlcNH_2_(6S)	0.0
**CS/DS Disaccharides**	**Disaccharides Produced (%) ^1^**
∆HexA-GalNAc	3.2
∆HexA-GalNAc(4S)	33.5
∆HexA-GalNAc(6S)	17.2
∆HexA(2S)-GalNAc(4S)	0.0
∆HexA(2S)-GalNAc(6S)	0.7
∆HexA-GalNAc(4S)(6S)	45.4
∆HexA(2S)-GalNAc(4S)(6S)	0.0

Abbreviations: ∆HexA, 4,5 unsaturated uronic acid; GlcNAc, *N*-acetylglucosamine; GlcNS, *N*-sulphated glucosamine; GlcNH_2_, glucosamine; GalNAc, *N*-acetylgalactosamine; 2S, 2-*O*-sulphate; 4S, 4-*O*-sulphate; 6S, 6-*O*-sulphate.

**Table 2:** HS Disaccharide analysis of ion-exchange purified cockle polysaccharide fractions. Data are presented as a percentage of the moles of unsaturated disaccharides produced by heparinase I, II, III digestion of the ion-exchange fractions (F1–F6).

HS Disaccharides	F1 (%)	F2 (%)	F3 (%)	F4 (%)	F5 (%)	F6 (%)
∆HexA-GlcNAc	4.6	28.0	84.4	31.9	9.8	6.8
∆HexA(2S)-GlcNAc	0.0	0.0	0.0	0.0	0.9	0.4
∆HexA(2S)-GlcNH_2_	0.0	0.0	0.0	0.1	0.2	0.0
∆HexA-GlcNAc(6S)	0.2	2.4	1.6	5.3	4.9	3.3
∆HexA(2S)-GlcNAc(6S)	30.4	10.9	0.0	0.0	3.1	4.6
∆HexA-GlcNS	0.7	8.6	12.9	28.4	10.4	5.9
∆HexA(2S)-GlcNS	16.6	25.2	0.5	12.8	24.4	34.0
∆HexA-GlcNS(6S)	4.9	10.9	0.6	15.9	19.4	16.4
∆HexA-GlcNH_2_(6S)	0.0	0.0	0.0	0.1	0.2	0.0
∆HexA(2S)-GlcNS(6S)	42.7	14.0	0.0	5.7	26.3	28.6
∆HexA(2S)-GlcNH_2_(6S)	0.0	0.0	0.0	0.0	0.3	0.1
Unsulphated	4.6	28.0	84.4	31.9	9.8	6.8
*N*-SO_3_	64.8	58.8	14.0	62.6	80.5	84.8
2-*O*-SO_3_	89.6	50.2	0.6	18.5	55.3	67.6
6-*O*-SO_3_	78.1	38.2	2.2	27.0	54.2	53.0
Total GAG disaccharides produced by heparinase digestion (μg)	0.01	0.002	0.01	0.05	0.29	0.06
Average sulphate per disaccharide	2.33	1.47	0.17	1.08	1.9	2.05

Abbreviations: ∆HexA, 4,5 unsaturated uronic acid; GlcNAc, *N*-acetylglucosamine; GlcNS, *N*-sulphated glucosamine; GlcNH_2_, glucosamine; GalNAc, 2S, 2-*O*-sulphate; 6S, 6-*O*-sulphate

**Table 3:** CS/DS Disaccharide analysis of ion-exchange purified cockle polysaccharide fractions. Data are presented as a percentage of the moles of unsaturated disaccharides produced by chondroitinase ABC digestion of the ion-exchange fractions (F1–F6).

CS/DS Disaccharides	F1 (%)	F2 (%)	F3 (%)	F4 (%)	F5 (%)	F6 (%)
∆HexA-GalNAc	70.6	72.4	86.4	8.8	3.5	3.6
∆HexA-GalNAc(4S)	23.1	26.7	13.6	89.9	56.8	35.0
∆HexA-GalNAc(6S)	3.8	0.2	0.0	1.4	3.7	4.3
∆HexA(2S)-GalNAc(4S)	0.0	0.0	0.0	0.0	0.0	0.0
∆HexA(2S)-GalNAc(6S)	0.0	0.0	0.0	0.0	1.8	3.9
∆HexA-GalNAc(4S)(6S)	2.5	0.8	0.0	0.0	34.2	53.2
∆HexA(2S)-GalNAc(4S)(6S)	0.0	0.0	0.0	0.0	0.0	0.0
Unsulfated	70.6	72.4	86.4	8.8	3.5	3.6
2-*O*-SO_3_	0.0	0.0	0.0	0.0	1.8	3.9
4-*O*-SO_3_	25.6	27.5	13.6	89.9	91.0	88.2
6-*O*-SO_3_	6.3	1.0	0.0	1.4	39.7	61.5
Total GAG disaccharides produced by ABC lyase digestion (μg)	0.002	0.006	0.006	0.026	0.285	0.086
Average sulfate per disaccharide	0.32	0.28	0.14	0.91	1.31	1.51

Abbreviations: ∆HexA, 4,5 unsaturated uronic acid; GalNAc, *N*-acetylgalactosamine; 2S, 2-*O*-sulphate; 4S, 4-*O*-sulphate; 6S, 6-*O*-sulphate

**Table 4:** HPAEC-PAD analysis of monosaccharides derived from unfractionated (crude) cockle polysaccharide and ion-exchange purified fractions (F1–F6). Samples (50 µg) were degraded to monosaccharides by treatment with TFA prior to high performance anion exchange chromatography with pulsed amperometric detection (HPAEC-PAD) analysis. The peaks observed were identified by comparison to the elution position of known monosaccharide standards. Data are presented as a percentage of the moles of monosaccharide produced by acid hydrolysis.

Monosaccharide	Crude (%)	F1 (%)	F2 (%)	F3 (%)	F4 (%)	F5 (%)	F6 (%)
Fuc	11.1	5.1	5.8	3.3	3.6	14.2	2.1
GalNH_2_	16.7	3	4.0	11.7	35.6	21.8	9.5
GlcNH_2_	9.9	3.9	2.1	11.9	10.4	14.5	3.6
Gal	19.1	2.3	3.0	11.0	14.4	20.1	8.8
Glc	35.2	73.5	57.4	52.9	30.0	10.4	50.4
Man	3.7	5.0	28.1	7.4	2.7	4.9	23.5
GlcA	4.3	1.0	0.7	1.9	3.3	14.1	2.1
IdoA	0.0	0.0	0.0	0.0	0.0	0.0	0.0

Abbreviations: Fuc, fucose; GalNH_2_, galactosamine; GlcNH_2_, glucosamine; Gal, galactose; Glc, glucose; Man, mannose; GlcA, glucuronic acid; IdoA, iduronic acid :
